# First-line high-dose therapy and autologous blood stem cell transplantation in patients with primary central nervous system non-Hodgkin lymphomas—a single-centre experience in 61 patients

**DOI:** 10.1007/s00277-021-04745-z

**Published:** 2022-01-04

**Authors:** T. Brezina, H. von Dewitz, T. Schroeder, S. Ullrich, K. Nachtkamp, G. Reifenberger, B. Malzkorn, M. Sabel, R. Haas, G. Kobbe

**Affiliations:** 1 Department of Oncology, University Hospital Zuerich, Zuerich, Switzerland; 2 Department of Internal Medicine A, Hospital Merheim, Cologne, Germany; 3 Department of Haematology, Medical Faculty, University Hospital Duesseldorf, Heinrich Heine University Duesseldorf, Duesseldorf, Germany; 4 Life Science Center for Statistics, Duesseldorf, Germany; 5 Department of Neuropathology, University Hospital Duesseldorf, Duesseldorf, Germany; 6 Department of Neurosurgery, University Hospital Duesseldorf, Duesseldorf, Germany

**Keywords:** Primary CNS lymphoma, High-dose chemotherapy, Hematopoietic stem cell transplantation, Methotrexate

## Abstract

Primary central nervous system non-Hodgkin lymphomas (PCNS-NHLs) are extranodal B-cell lymphomas with poor prognosis. The role of high-dose therapy (HDT) followed by autologous blood stem cell transplantation (ASCT) as first-line therapy is still not clear. We retrospectively collected long-term follow up data of 61 consecutive patients with PCNS-NHL at the University Hospital Düsseldorf from January 2004 to December 2016. Thirty-six patients were treated with conventional chemoimmunotherapy (cCIT) only (CT-group). Seventeen patients received an induction cCIT followed by HDT and ASCT. In the CT-group, the overall response rate (ORR) was 61% (CR 47%, PR 14%), and there were 8% treatment-related deaths (TRD). Progression-free survival (PFS) was 31.8 months, and overall survival (OS) was 57.3 months. In the HDT-group, the ORR was 88% (59% CR, 29% PR), and there were 6% TRD. Median PFS and OS were not reached at 5 years. The 5-year PFS and OS were 64.7%. After a median follow up of 71 months, 10 patients (59%) were still alive in CR/PR following HDT and ASCT, one patient was treated for progressive disease (PD), and 7 had died (41%, 6 PD, 1 TRD). All patients achieving CR prior to HDT achieved durable CR. In the CT-group, 8 patients (22%) were alive in CR/PR after a median follow-up of 100 months. Twenty-eight patients died (78%, 24 PD, 2 TRD, 2 deaths in remission). In the univariate analysis, the HDT-group patients had significantly better PFS (not reached vs 31.8 months, *p* = 0.004) and OS (not reached vs 57.3 months, *p* = 0.021). The multivariate analysis showed HDT was not predictive for survival. Treatment with HDT + ASCT is feasible and offers the chance for long-term survival with low treatment-related mortality in younger patients. In this analysis, ORR, PFS and OS were better with HDT than with conventional cCIT alone. This result was not confirmed in the multivariate analysis, and further studies need to be done to examine the role of HDT in PCNSL.

## Introduction

Primary central nervous system lymphomas (PCNSLs) are rare extranodal non-Hodgkin lymphomas occurring exclusively in the central nervous system. They represent approximately 2% of all primary central nervous tumours and approximately 1% of all extranodal lymphomas [1, 2]. The most common histological subtype is the diffuse large B-cell lymphoma, and rarely Burkitt, Hodgkin or T-cell lymphoma [2, 3]. PCNSL respond well to chemotherapy entering the central nervous system and to radiotherapy, but prognosis remains poor compared to their nodal counterparts. A standard treatment for this rare hematologic disease remains to be established. Historically, whole-brain radiotherapy (WBRT) was used to treat PCNSL. High response rates were achieved, yet overall survival (OS) rates remained low, as most patients suffered a relapse within a year after WBRT [4–6]. In addition, side effects of radiotherapy are substantial. In need of a more effective therapy, methotrexate (MTX) was incorporated into the upfront therapy regimen. With single-agent MTX, overall response rates (ORRs) of 52–97% with stable and durable remissions have been achieved [7–11]. The combination of high-dose MTX with WBRT has proven to be more effective with a 5-year OS as high as 37% and median OS of 33 months [11, 12]. Escalation of the induction therapy to the combination of MTX with other cytotoxic agents prior to a consolidation radiation therapy has been shown to have an additional positive impact on the disease control, further improving the OS to 37–60 months [13–17]. Due to a high incidence of neurotoxicity after a combination of radiotherapy and high-dose MTX, radiotherapy is generally not recommended as a primary treatment. Omitting radiotherapy from the upfront regimen has had no negative impact on OS [7, 18].

Based on studies on peripheral lymphomas, the anti-CD20 antibody, rituximab, has been added to the upfront therapy of PCNSL. In an ongoing phase 3 study, the addition of rituximab and thiotepa to MTX and cytarabine was associated with a significantly longer OS and PFS [19]. In contrast to these findings, another prospective study did not show a clear benefit of rituximab added to the front-line therapy [20]. Due to a high efficacy in peripheral lymphomas, rituximab is generally added to an MTX-based induction chemotherapy in patients with PCNSL.

Over the years, different consolidating strategies have been investigated. High-dose therapy (HDT) with autologous blood stem cell transplantation (ASCT) has been broadly accepted as an effective therapy to consolidate the response achieved by induction therapy. In two phase 2 studies, an ORR of 90% with a median PFS over 74 months were achieved with the combination of induction chemotherapy and HDT with ASCT [21, 22]. In another multicentre phase 2 study, HDT with ASCT without WBRT was shown to induce high rates of disease control in young patients with PCNSL. The overall response rate was 91%, the median OS was 104 months, and there were no treatment-related deaths [23]. The treatment-related mortality reported in phase 2 studies on HDT with ASCT varied between 0 and 12% [22, 24, 25]. Recent phase 2 studies directly compared consolidation therapy with HDT and ASCT with WBRT. Both studies have shown HDT and WBRT to be an effective consolidation in patients with PCNSL. With both strategies, similar PFS and OS rates could be achieved. Consistent with historic studies, the negative impact on cognitive functions was higher in the WBRT group [24, 26]. These studies showed that consolidation HDT and ASCT is a very effective approach with tolerable toxicity in young patients. The efficacy of a consolidation strategy with high-dose chemotherapy and ASCT versus a non-myeloablative consolidation with conventional chemotherapy is currently being tested in several randomized trials (NCT01511562, NCT02531841).

The main objective of this retrospective study was the assessment of “real-world” response to either conventional therapy or to HDT with ASCT and their impact on the PFS and OS among patients treated at the Department of Hematology, Oncology and clinical Immunology of the University Hospital Duesseldorf.

## Methods

### Study design

This is a retrospective, single-centre, registry-based analysis covered by a positive vote of the ethics committee of the Heinrich Heine University Duesseldorf. Patients were eligible for inclusion if they were aged 18 or older with newly diagnosed PCNSL. The diagnosis was suggested through magnetic resonance imaging, and definitive diagnosis of PCNSL was made by a histopathological examination of a tumour specimen obtained through a stereotactic biopsy or a tumour resection. At the initial diagnosis, all patients underwent staging with computed tomography of the chest, abdomen and pelvis. All patients with involvement outside the central nervous system at the time of diagnosis were excluded from this study. Data on patients’ characteristics, treatment and outcome were retrospectively collected from patients’ charts and the hospital information system.

### Statistical analysis

Descriptive statistics were used to summarize patient characteristics. Primary endpoints were overall survival, defined as the time from the start of therapy to death from any cause, as well as progression-free survival, defined as the time from the start of therapy to disease relapse or progression or death. Secondary endpoints were ORR, defined as the proportion of patients who achieved a partial or complete response to therapy, and treatment-related mortality (TRM), defined as death in a direct relation to treatment and in the absence of prior relapse or progression. The Kaplan–Meier methodology and log-rank test were used to examine OS and PFS according to patients’ characteristics. Cox’s regression model was used to determine the clinical predictors for OS and PFS. The factors examined were the first-line therapy (HDT and cCIT), age, international prognostic score, the use of radiotherapy and the use of rituximab. Median follow-up of all surviving patients was 85.5 months (range 57–196) at February 2021.

## Results

We collected data of 61 patients with PCNS-NHL who were diagnosed and treated at the University Hospital Düsseldorf between January 2004 and December 2016. The cohort consisted of sixty-one patients with a median age of 64 years (range 29–80). Fifty-nine per cent of the patients were male. Patients in the high-dose therapy (HDT) group were younger when compared to patients in the conventional therapy (CT) group (54.6 years vs. 66.5 years, *p* < 0.001). Patients’ characteristics are summarized in Table 1.

**Table 1:** Patient characteristics at diagnosis

	HDT group	CT group
Male	13 of 17 (76%)	20 of 36 (56%)
Age > 60 years	6 of 17 (35%)	28 of 36 (78%)
ECOG 0–1	12 of 16 (75%)	23 of 33 (70%)
LDH elevation	3 of 15 (20%)	8 of 33 (24%)
IPI score 0–2	11 of 15 (75%)	20 of 33 (61%)
MSKCC score 0–2	13 of 15 (87%)	23 of 33 (70%)
Multiple Lesions (> 1)	7 of 17 (41%)	20 of 35 (57%)
Diagnosis by:		
- biopsy	17 of 17 (100%)	21 of 35 (60%)
- partial resection	0 of 17 (0%)	7 of 35 (20%)
- complete resection	0 of 17 (0%)	7 of 35 (20%)
Histologic subtype		
- diffuse large B-cell lymphoma	17 of 17 (100%)	34 of 35 (97%)
- anaplastic B-cell lymphoma	0 of 17 (0%)	1 of 35 (3%)
CD-20 positivity	13 of 13 (100%)	24 of 33 (73%)
Radiotherapy	0 of 17 (0%)	2 of 36 (6%)

**Fig. 1:**
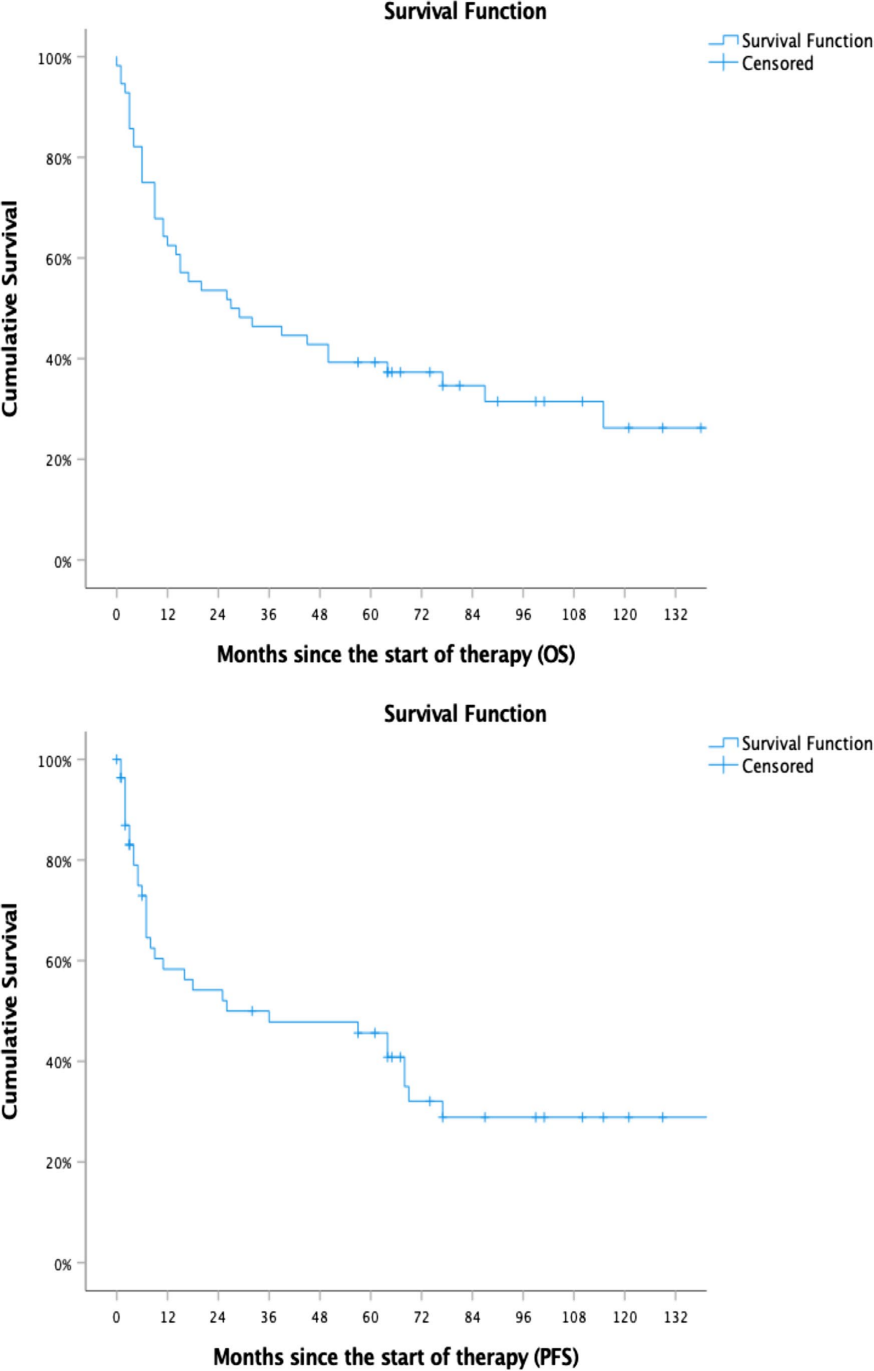
PFS and OS comparing both groups

In the CT-group, 36 patients were treated with conventional chemo-immunotherapy (cCIT). Two patients in this group received consolidating whole brain radiotherapy. In the HDT-group, 17 patients received induction chemo-immunotherapy followed by HDT with ASCT. Three patients were treated solely with radiotherapy, and five patients received best supportive care only. Patients that received only radiotherapy or best supportive care were not included in further analyses (Fig. 1).

### High-dose chemotherapy group

Induction chemo-immunotherapy followed by consolidation therapy consisting of HDT and ASCT was performed in 17 patients. The induction therapy preceding HDT consisted mainly of rituximab and high-dose MTX (median 3 cycles; range 2–6 cycles), high-dose cytarabine and thiotepa (median 2 cycles; range 1–4 cycles). All patients achieved an objective response after induction therapy: five patients (29%) achieved complete remission (CR) and 12 patients (71%) achieved partial remission (PR). The conditioning regimen consisted of BCNU and thiotepa, with or without rituximab in 14 patients (82%). Three patients (18%) received BCNU, etoposide, cytarabine and melphalan, with or without rituximab as a conditioning regimen. The details on HDT with ASCT are shown in Table 2.

**Table 2:** Conditioning regimens in high-dose therapy group

Conditioning regimen	n (%)
Rituximab/BCNU/thiotepa	7 (41%)
BCNU/thiotepa	7 (41%)
Rituximab/BCNU/etoposide/cytarabine/melphalan	2 (12%)
BCNU/etoposide/cytarabine/melphalan	1 (6%)

**Fig. 2:**
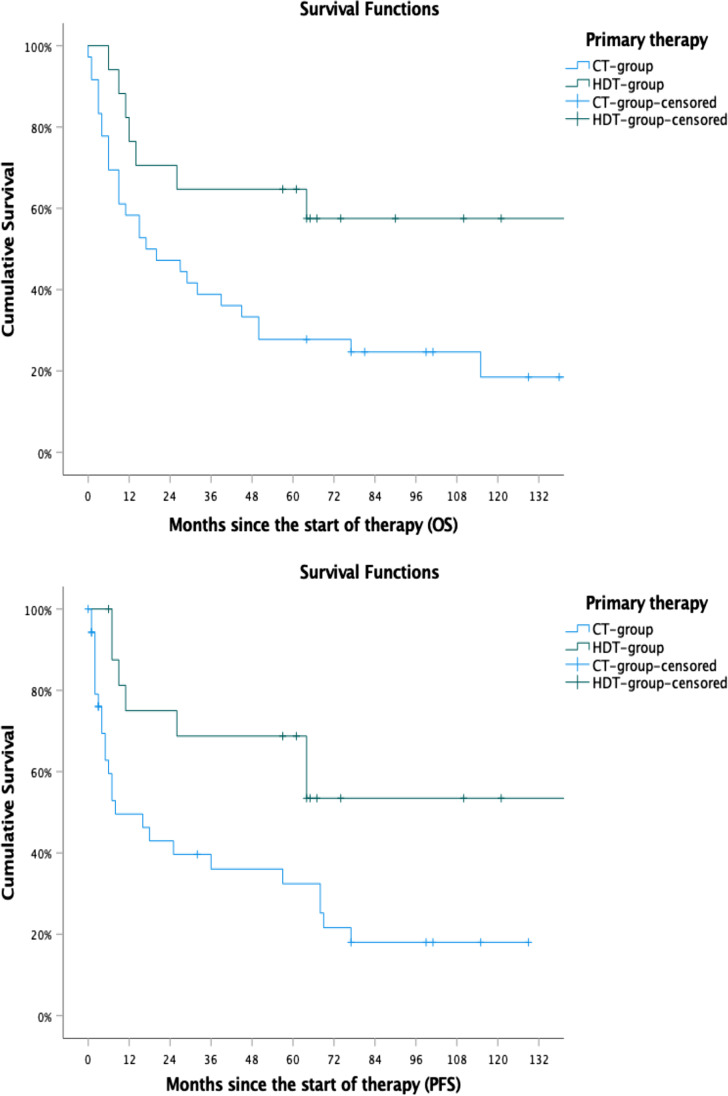
PFS and OS of the whole cohort

With a median follow-up of 71 months (range 57–147), the ORR in the HDT group was 88%, and there was 1 treatment-related death due to sepsis (6%). Seven patients died of progressive disease after HDT (41%). Median PFS and OS at 5 years after diagnosis were not reached in the HDT group. Estimated 5-year PFS was 64.7% (95% CI, 46–91%), and estimated 5-year OS was 64.7% (95% CI, 42–87%: Fig. 2). After HDT with ASCT, 10 patients (59%) were alive at the last recorded follow-up. Nine patients (53%) were in CR or PR and one patient was treated with radiotherapy due to progressive disease. Seven patients had died (41%, 6 progressive disease, 1 treatment related death). All patients that achieved CR prior to HDT remained in CR at the last documented follow-up.

### Conventional chemotherapy group

Thirty-six patients were treated with conventional chemo-immunotherapy. Therapy regimens varied in this cohort. Twenty-eight patients (78%) received single-agent MTX with or without rituximab. A median of 3 cycles (range 2–8) of high-dose MTX was administered. Eight patients (22%) received other regimens listed in Table 3. Two patients received consolidating radiotherapy.

**Table 3:** Chemotherapy regimens in conventional chemotherapy group

Chemotherapy in CT arm	*n* (%)
Methotrexate	11 (31%)
Rituximab/methotrexate	17 (47%)
Rituximab/methotrexate/cytarabine	3 (8%)
Rituximab/methotrexate/procarbazine/vincristine	2 (6%)
Rituximab/ifosfamid	1 (3%)
Rituximab/methotrexate/ifosfamid	1 (3%)
Rituximab/cytarabine	1 (3%)

The ORR in the CT group was 61%. Seventeen patients (47%) achieved CR, and five patients (14%) achieved PR. Two patients (6%) had treatment-related deaths (due to sepsis). PFS was 31.8 months, and OS was 57.3 months, with a median follow-up in surviving patients of 100 months (range 64–196). The survival curves are shown in Fig. 2. In the CT-group, eight patients (22%) were alive at the last documented follow-up. Only 3 patients (8%) in the CT-group achieved a durable CR. Five patients (14%) suffered from progressive disease. Of those, 2 underwent salvage HDT with ASCT, two received a salvage radiotherapy, and one received salvage chemoimmunotherapy. Twenty-four patients treated with conventional chemotherapy (67%) died of disease progression, two patients died in remission of unrelated causes (6%), and there were two treatment-related deaths (6%, due to sepsis).

### Outcome

**Fig. 3:**
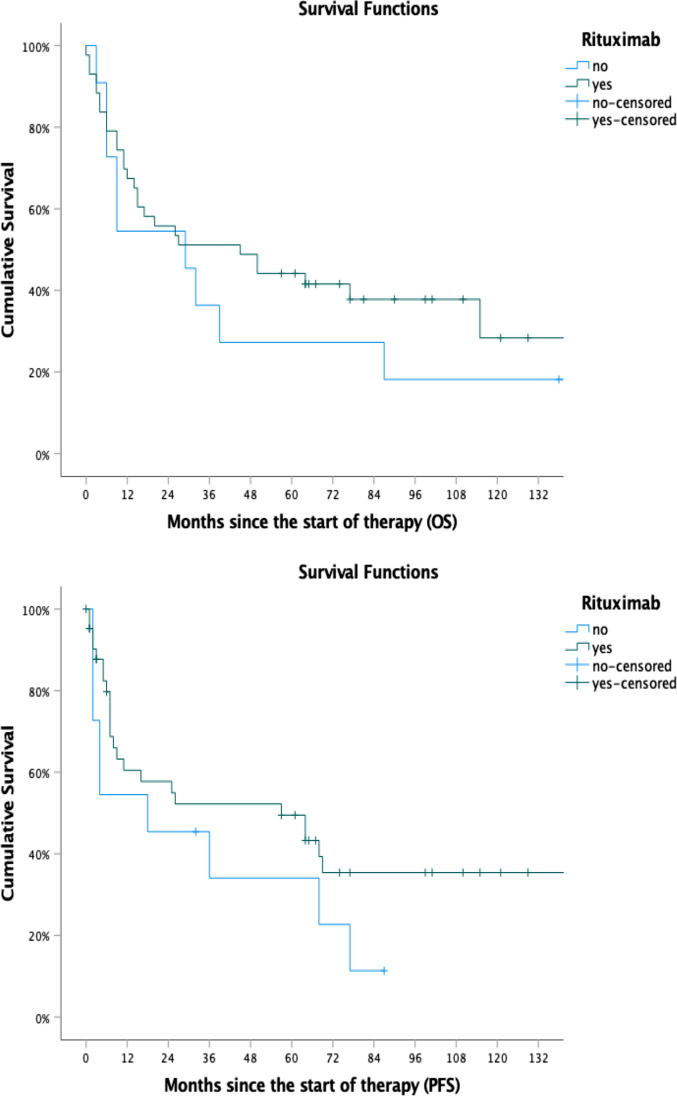
Progression-free survival and overall survival in patients treated with or without rituximab

For the whole patient cohort, PFS was 48.1 months and OS was 76.1 months (Fig. 1). Compared to the CT group, PFS was significantly longer in the HDT group (not reached vs 32.7 months, *p* = 0.004). The OS was also found to differ significantly between the HDT group and the CT group (not reached vs 57.3 months, *p* = 0.021). Patients in the HDT group were younger when compared to patients in the CT group (54.6 years vs 66.5 years, *p* < 0.001). The survival curves for both groups are shown in Fig. 2. The addition of rituximab to conventional chemotherapy did not have an impact on PFS or OS in either group as shown in Fig. 3. When age, the use of rituximab, the use of radiotherapy, the international prognostic score and the use of HDT were included in a multivariate model, HDT was not an independent predictive parameter for PFS (*p* = 0.260; HR 0.51; 95% CI 0.15–1.65) and OS (*p* = 0.148; HR 0.43; CI 0.13–1.35). Although the use of HDT did not reach a statistical significance in the multivariate analysis, the hazard ratios of 0.43 and 0.51 for OS and PFS, respectively, suggest a favourable outcome of the HDT group in comparison with the cCIT group. The results of the multivariate analysis are shown in Table 4.

**Table 4:** Multivariate analysis for factors affecting progression-free and overall survival

	PFS			OS		
Factor	HR	95% CI	*p*-value	HR	95% CI	*p*-value
First line therapy	0.51	0.15–1.65	0.260	0.43	0.13–1.35	0.148
Age	1.44	0.16–13.32	0.748	1.44	0.16–13.15	0.748
IPI Score	1.57	0.87–2.83	0.136	1.29	0.74–2.25	0.374
Radiotherapy	0.80	0.29–2.18	0.658	0.92	0.34–2.52	0.874
Rituximab	0.50	0.21–1.19	0.118	0.79	0.34–1.84	0.589

## Discussion

Treatment of patients with PCNSL remains a challenge. The best standard treatment for PCNSL has not yet been established. Due to the low incidence of PCNSL, the evidence for effective induction and consolidation therapy is limited. Only a few phase II trials have shown benefit from HDT. As most of the trials included only a small number of patients, the role, feasibility and efficacy of upfront HDT with ASCT still remain uncertain.

WBRT has been shown to be an effective consolidation therapy, but is generally omitted from first-line therapy, due to high rates of neurotoxicity. HDT with ASCT may be an alternative to consolidating irradiation. In young patients, HDT has become a standard upfront therapy, although clear evidence in favour of HDT over consolidation with standard chemotherapy is missing.

In our study, induction therapy, consisting of cCIT, led to CR in 29% and an ORR of 100% among patients in the HDT group. High-dose therapy further improved the CR rate to 59%. Interestingly, all patients that achieved CR prior to HDT had a better outcome than those achieving CR after the HDT. This suggests the importance of an effective induction therapy prior to proceeding to a definitive consolidation therapy. The majority of our patients received a BCNU and thiotepa-based HDT. This conditioning regimen proved to be more effective than other regimens commonly used in peripheral lymphomas.

In recent years, randomised trials and retrospective studies have evaluated the role of HDT. In one prospective study, HDT was applied after induction therapy with HD-MTX. The addition of cytarabine and thiotepa as a mobilisation chemotherapy to the induction regimen led to a high ORR of 80% prior to HDT with thiotepa and carmustin. The 5-year OS in this study was 70% and the median OS as high as 104 months [23]. Another phase II study showed similar effectivity of MTX-based induction therapy and thiotepa and carmustin-based HDT as the 5-year OS was 79% [22]. The addition of cyclophosphamide to the HDT therapy with carmustin und thiotepa and intensification of the MTX-based induction treatment with procarbazine and vincristine was associated with an improvement of the CR rate to nearly 66% and ORR to 97%. After HDT, the CR rate rose to 77%, and the 2-year OS was 81% [25]. In another phase 2 trial, patients received an induction therapy consisting of high-dose MTX, high-dose cytarabine and thiotepa. The induction therapy was followed by a BCNU and thiotepa-based HDT. The treatment with HDT led to a further improvement of the ORR (62% prior to HDT and 84% after HDT). The 3-year OS was 77% in the group that underwent HDT with ASCT [27]. These values correlate well with our results, which showed high ORR and survival rates in the HDT-group.

In our retrospective study, most of the patients in the CT group received single-agent MTX. After a median of 3 cycles, which were mostly combined with rituximab, the ORR was 62%. These results are consistent with previously published studies that examined the role of MTX in the treatment of PCNSL. Single-agent MTX achieved an ORR ranging from 52 to 97%. PFS from 7 to 13 months and OS from 37 to 54 months were reported [7–10]. The addition of rituximab to a chemotherapy backbone did not lead to a significant improvement in PFS and OS [20, 28]. In the CT cohort of our study, we observed similar findings (PFS was 32.7 months and OS was 57.3 months). Moreover, the addition of rituximab to the conventional chemotherapy or induction therapy prior to HDT had no effect on the PFS and OS. Furthermore, there was relatively low TRM in both groups (8% in the CT group, 6% in the HDT group), comparable to the TRM reported in previous studies [22, 24, 25].

The findings of our retrospective analysis of patients with PCNSL treated at our institution show in the univariate analysis a benefit of consolidation HDT with ASCT in eligible patients compared to standard chemotherapy. Patients in our study that received HDT achieved significant longer PFS and OS which is consistent with previously published studies. OS was longer in the HDT-group despite the fact that many patients in the CT-group with progression or relapse proceeded to an effective salvage therapy. In the multivariate analysis, the effect of primary HDT on the OS and PFS did not reach statistical significance. The hazard ratio for PFS and OS yet indicate a tendency for a better outcome with a first-line HDT as opposed to a primary cCIT.

Our study has a number of limitations that could have had an impact on the results reported. First, it is a retrospective study with a limited number of patients included. Second, the patients’ characteristics in both cohorts were very heterogenous, and patients in the HDT group were significantly younger when compared to the CT group. Third, the induction and consolidation therapy lacked standardization.

In conclusion, intensive cCIT induction followed by HDT with ASCT is an effective therapy achieving high cure rates with low treatment-related mortality in selected patients with PCNS-NHL. Further randomized comparative studies are needed to define the role of HDT and ASCT in the treatment for PCNS-NHL.

## Data Availability

The datasets generated during and/or analysed during the current study are available from the corresponding author on reasonable request.

## Code availability

The final DOI will be cited in the manuscript.
